# Active Immunoprophylaxis and Vaccine Augmentations Mediated by a Novel Plasmid DNA Formulation

**DOI:** 10.1089/hum.2018.241

**Published:** 2019-04-04

**Authors:** Nina N. Schommer, Jacklyn Nguyen, Bryan S. Yung, Katherine Schultheis, Kar Muthumani, David B. Weiner, Laurent Humeau, Kate E. Broderick, Trevor R.F. Smith

**Affiliations:** ^1^ Inovio Pharmaceuticals, Inc., Plymouth Meeting, Pennsylvania; ^2^ The Wistar Institute of Anatomy and Biology, Philadelphia, Pennsylvania.

**Keywords:** gene delivery, plasmid DNA, therapeutics

## Abstract

Plasmid DNA (pDNA) gene delivery is a highly versatile technology that has the potential to address a multitude of unmet medical needs. Advances in pDNA delivery to host tissue with the employment of *in vivo* electroporation (EP) have led to significantly enhanced gene expression and the recent demonstration of clinical efficacy with the platform. Building upon this platform, this study reports that enzyme-mediated modification of the muscle tissue extracellular matrix structure at the site of pDNA delivery operates in a synergistic manner with EP to enhance both local and systemic gene expression further. Specifically, administration of chondroitinase ABC (Cho ABC) to the site of intramuscular delivery of pDNA led to transient disruption of chondroitin sulfate scaffolding barrier, permitting enhanced gene distribution and expression across the tissue. The employment of Cho ABC in combination with CELLECTRA^®^ intramuscular EP resulted in increased gene expression by 5.5-fold in mice and 17.98-fold in rabbits. The study demonstrates how this protocol can be universally applied to an active prophylaxis platform to increase the *in vivo* production of functional immunoglobulin G, and to DNA vaccine protocols to permit drug dose sparing. The data indicate the Cho ABC formulation to be of significant value upon combination with EP to drive enhanced gene expression levels in pDNA delivery protocols.

## Introduction

Plasmid DNA (pDNA)-based gene transfer has emerged as an attractive platform to target infectious disease,^1–6^ cancer,^7–14^ tissue repair, and regeneration.^15,16^ Advances in this technology, specifically in the intramuscular delivery of pDNA by *in vivo* electroporation (EP), have permitted robust expression of transgenes, offering a viable alternative platform to the use of viral vectors.^17^ For the production of systemic levels of the expressed gene, the skeletal muscle is an ideal target tissue for the delivery of pDNA for the following reasons.^18,19^ The skeletal muscle is highly vascularized, possesses low lysosomal and DNase content, and is highly accessible and abundant throughout the vertebrate body. Each muscle fiber represents a single cell with a large volume and a syncytium consisting of multiple nuclei. The stability and terminal differentiation state of the skeletal muscle cells will prevent the loss of pDNA through cell division. However, many hurdles hinder the efficient delivery of pDNA into the myocyte. The size and negative charge of pDNA impede the efficient passage through the cell wall. In response, several enabling platforms have been employed to achieve efficient gene delivery to the muscle, the most impactful platform being *in vivo* EP, which involves a transient disruption of the cell membrane to allow passage of pDNA into the cell, improving pDNA delivery efficiency by a factor of 100- to 1,000-fold over naked pDNA delivery alone.^17,20^

The skeletal muscle extracellular matrix (ECM) surrounding each muscle fiber represents a further barrier, which acts to hinder myocyte transfection efficiency. Glycosaminoglycans (GAGs), such as hyaluronan and chondroitin sulfate, are ubiquitous in the muscle ECM, forming branch-like structures that may impede the injected pDNA achieving proximity to and interaction with the myocyte.^21^ Previously, it was shown that pretreatment of muscle tissue with hyaluronidase—an enzyme that catalyzes the hydrolysis of the GAG hyaluronan—before EP-assisted pDNA delivery resulted in significant enhanced gene expression across the tissue.^22–24^ Chondroitin sulfate, which consists of alternating sulfate-substituted units of N-acetylgalactosamin and glucuronic acid, can be reversibly disrupted by the enzyme chondroitinase. Here, it was hypothesized that the transient enzymatic disruption of chondroitin sulfate in the muscle ECM increases the accessibility of pDNA to the muscle fibers, leading to an elevated transfection rate of myocytes and increased levels of gene expression. To this purpose, clinical grade chondroitinase ABC (Cho ABC) was applied to EP-assisted pDNA delivery protocols, and the effect on local and systemic gene expression levels was assessed in mouse and rabbit models. Importantly, an advanced protocol is described in which pDNA and Cho ABC are co-formulated, avoiding the requirement to pretreat the muscle with the enzyme. In summary, a clinically relevant pDNA delivery protocol is described, which leads to enhanced levels of gene expression.

## Methods

### Plasmid constructs

The DNA plasmid pMERS was designed, as previously described.^2,25^ pMERS encodes the light and heavy chains for the full-length anti-MERS envelope glycoprotein monoclonal antibody. The light and heavy chains are expressed as a single mRNA transcript and then cleaved post-translationally at a porcine teschovirus-1 2A (P2A) cleavage site. A furin cleavage site (RGRKRRS) was also included to ensure complete removal of the P2A from the final *in vivo* produced antibody. pNP encodes the full-length nucleoprotein (NP) from influenza A (H1N1, A/Puerto Rico/8). The *MERS* and *NP* transgenes were cloned into a modified mammalian expression vector (pVax1, Genscript) under the control of the human cytomegalovirus promoter. pRFP reporter gene plasmid was purchased from Aldevron (Fargo, ND).

### Enzymes

Cho ABC was purified from *Proteus vulgaris* (Amsbio, Cambridge, MA). HYLENEX recombinant is a purified preparation of the enzyme recombinant human hyaluronidase, produced by genetically engineered Chinese hamster ovary cells containing a DNA plasmid encoding for a soluble fragment of human hyaluronidase (PH20; Halozyme Therapeutics, San Diego, CA).

### Animals

All animals were purchased from Charles River Laboratories (Wilmington, MA). All animals were housed and treatments performed at Acculab, Inc., vivarium (San Diego, CA). The ethical study committee protocol number for each species is provided in parentheses. Female Balb/c mice were group housed four mice per cage (CalMI2–047). Female New Zealand White (NZW) rabbits (9 weeks old) were single housed (CalMI2–045). Female Hartley guinea pigs (10 weeks old) were group housed four guinea pigs per cage (CalMI2–043). Animal husbandry was in accordance to the guidelines of the Institutional Care and Use Committee.

### Intramuscular injection of enzymes and pDNA with EP

#### Mice

All animals were sedated with isoflurane inhalation and shaved at the treatment side prior to treatment. Unless otherwise stated, the left leg tibialis anterior (TA) muscle of each mouse was pretreated with 30 μL of 2.5 IU/mL Cho ABC 30 min before pDNA delivery and EP. Mice in the control groups received phosphate-buffered saline (PBS) as a pretreatment. pDNA was administered at the defined dose in 30 μL PBS intramuscularly, and EP was performed after the injection. EP was applied to the injection site using a CELLECTRA^®^-3P (3 mm electrodes) device (Inovio Pharmaceuticals, Inc., Plymouth Meeting, PA). Two 0.1 A constant current wave pulses were applied to the muscle site. Each pulse was 52 ms long, with a 0.2 s delay between pulses and 3 s between each set of pulses.

#### Rabbits

After sedation with isoflurane, animals were shaved at the treatment site quad muscle. pMERS (1 mg per site) in 1 mL formulation was administered to the left rabbit quad muscle at two treatment sites. EP was applied to the site of injections in the skeletal muscle using a CELLECTRA^®^-5P device. Three pulses were applied, and the current strength was 0.5 A. Each pulse was 52 ms long, with a 1 s delay between pulses.

### Human immunoglobulin G quantification enzyme-linked immunosorbent assay

Ninety-six-well plates were coated at 4°C with goat anti-human immunoglobulin G (anti-hIgG) Fc fragment antibody (Bethyl Laboratories, Montgomery, TX; 10 μg/mL). After washing with 1 × PBS with 0.05% Tween, plates were blocked (10% fetal bovine serum [FBS]/PBS) for 1 h at room temperature. Serum samples and standard dilutions were added in duplicate to the assay plate for 2 h at room temperature. The assay standard was a purified hIgGκ monoclonal antibody (Bethyl Laboratories), and dilutions began at a concentration of 500 ng/mL (1:2 dilutions). Samples and standards were incubated for 1 h at room temperature. After washing, a secondary antibody (goat anti-human IgG kappa light chain horseradish peroxidase [HRP]; Bethyl Laboratories) was added to the plates. After incubation for 1 h at room temperature, plates were washed, and the 3,3′,5,5′-tetramethylbenzidine (TMB) substrate (KPL, Gaithersburg, MD) was added for development. The reaction was stopped by TMB stop solution (KPL), and optical density (OD) values at 450 nm were read on a plate reader (Molecular Devices, San Jose, CA).

### MERS antigen-binding enzyme-linked immunosorbent assay

Ninety-six-well plates (Nunc/Thermo Fisher Scientific, Waltham, MA) were coated with 0.3 μg/mL MERS-CoV Spike protein S1 (SinoBiological, Inc., Beijing, P.R. China) overnight at 4°C. After washing with 1 × PBS with 0.05% Tween, plates were blocked (10% FBS/PBS) for 1 h at room temperature. Pre-diluted serum samples (1:30) were added to row A. Serum serial dilutions (1:3; 50 μL) were performed on assay plates (Nunc/Thermo Fisher Scientific). Plates were incubated for 2 h at room temperature. Plates were washed, and secondary antibody (goat anti-human IgG kappa light chain HRP; Bethyl Laboratories) was added. After incubation for 1 h at room temperature, plates were washed, and the TMB substrate (KPL) was added for development. The reaction was stopped by TMB stop solution (KPL), and OD values at 450 nm were read on a plate reader (Molecular Devices).

### Influenza A PR8/34 antigen-binding enzyme-linked immunosorbent assay

To measure antibody responses against influenza NP antigen, 96-well pates were coated at 4°C with influenza A PR8/34 recombinant protein (0.3 μg/mL; SinoBiological, Inc.). After washing with 1 × PBS with 0.05% Tween, plates were blocked for 1 h at room temperature. Serum samples were diluted 1:20 in PBS, and serial (1:3) dilutions were made. The serum samples were added to and incubated on the assay plates (Nunc/Thermo Fisher Scientific) for 2 h at room temperature. After washing, a detection antibody (goat anti-mouse IgG HRP antibody; Bethyl Laboratories) was added to the plates. After incubation for 1 h at room temperature, plates were washed, and the TMB substrate (KPL) was added for development. The reaction was stopped by TMB stop solution (KPL), and OD values at 450 nm were read on a plate reader (Molecular Devices).

### Splenic lymphocyte preparation and interferon-gamma enzyme-linked immunospot assay

Splenic lymphocyte preparation was performed, as described previously.^26^ Briefly, spleens were homogenized in 5 mL R10 media (RPMI medium containing 10% FBS; Thermo Fisher Scientific) using the gentle MACS Octo Dissociater (Miltenyi Biotec, Auburn, CA). After filtrating steps, splenocytes were re-suspended in ACK lysis buffer to remove erythrocytes. Following the centrifugation steps, the cell pellet was re-suspended in R10 media, and cell numbers were counted in a VI-CELL XR cell counter (Beckman Coulter, Indianapolis, IN). To measure the number of cells secreting interferon gamma (IFN-γ), a mouse IFN-γ enzyme-linked immunospot (ELISpot) assay kit (Mabtech, Inc., Cincinnati, OH) was used according to the manufacturer's instructions. Briefly, plates were coated with the immobilized cytokine-specific monoclonal capture antibody and incubated at 4°C overnight. The plates were washed with PBS and blocked for 1 h with R10 media. The freshly prepared cell suspensions (2 × 10^5^ cells/100 μL) were added to the wells in the presence of NP55-69 and NP147-155 peptides (10 μg/mL; GenScript, Piscataway, NJ). Concanavalin A (2 μg/mL; Sigma–Aldrich, St. Louis, MO) was used as a positive control, and media was used as a negative control. After incubation for 20 h at 37°C at 5% CO_2_, plates were developed according to the manufacturer's instructions. Spot-forming cells were detected and analyzed using the Immunospot system and software (CTL, Cleveland, OH).

### Reporter gene plasmid and imaging

Mice were sacrificed under standard institutional protocol. Left and right hind limbs were dissected, and the skin was removed. The intensity of the plasmid encoding red fluorescent protein (RFP) TurboRFP in the TA muscle was measured at 593 nm by a fluorescent imaging system (ProteinSimple, San Jose, CA) and quantified by Adobe Photoshop CC2017.

### Histology

Mice were sacrificed, left and right hind limbs were dissected, and the skeletal muscles were isolated for hematoxylin and eosin (H&E) staining. H&E staining and scanning of the slides were performed at Reveal Biosciences (San Diego, CA). Analysis of the slides was performed using the software CaseViewer.

### Immunofluorescence staining of tissue chondroitin sulfate

For immunofluorescence staining, Hartley guinea pigs were sacrificed, and the left and right TA muscles were dissected and fixed in 4% paraformaldehyde. After incubation in 30% sucrose in PBS and freezing steps, tissue sections (9 μm) were incubated with Cho ABC (2.5 IU/mL) for 15 min at 37°C. Chondroitin sulfate was detected by using a mouse monoclonal anti-chondroitin sulfate antibody (CS-56, Abcam, MA) and a goat anti-mouse IgG Alexa Fluor^®^ 488 antibody (Abcam, Cambridge, MA). Nuclei were stained with DAPI Fluoromount G (SouthernBiotech, Birmingham, AL).

### Immunofluorescence staining of tissue hyaluronan

Mice were sacrificed, and TA muscles were dissected and fixed in 4% paraformaldehyde. After incubation in 30% sucrose in PBS and freezing steps, tissue sections (9 μm) were incubated with Cho ABC (2.5 IU/mL) or hyaluronidase (150 IU/mL) for 3 h at 37°C. Hyaluronan was detected using biotinylated HABP and streptavidin, as well as an Alexa Fluor^®^ 555 secondary antibody (Abcam). Nuclei were stained with DAPI Fluoromount G (Southern Biotech).

### Agarose gel electrophoresis

Molecular weight and conformation of pDNA were determined by agarose gel electrophoresis (1% agarose, Embi Tec, San Diego, CA; TAE buffer). The ladder for supercoiled pDNA (2–10 kb) was purchased at New England Biolabs (Rowley, MA). Electrophoresis was run at 75 V for 80 min. Analysis was performed by Gene Sys Software.

### Statistical analysis

Statistical analysis was performed using GraphPad Prism v7.0 (GraphPad Software, Inc., San Diego, CA). Data are expressed as the mean ±standard error for each group. To compare group statistics, Mann–Whitney tests were performed.

## Results

### pDNA delivery into Cho ABC–treated muscles results in enhanced local gene expression in mice

**Figure 1.**
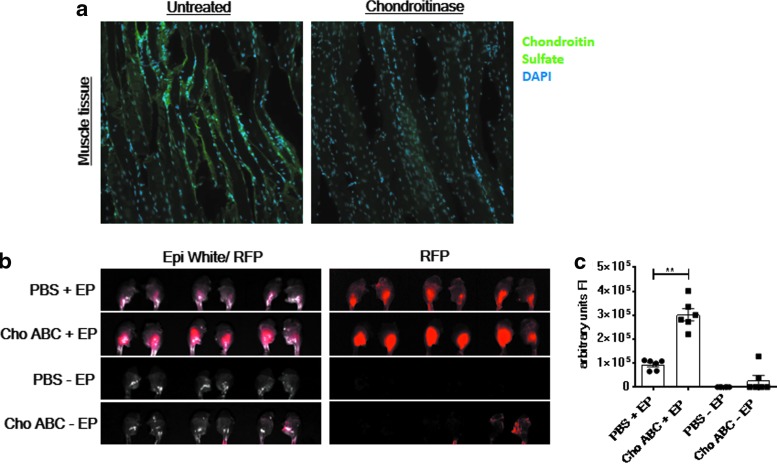
Chondroitinase treatment of skeletal muscle disrupts chondroitin sulfate matrix and permits enhanced local gene expression after electroporation (EP)-assisted delivery of plasmid DNA (pDNA). **(a)** Histological analysis of chondroitin sulfate signal in guinea pig muscle tissue after treatment with chondroitinase ABC (Cho ABC). Left quadricep muscles of Hartley guinea pigs were dissected and immediately fixed, and 9 μm sections were incubated with 2.5 IU/mL Cho ABC or left untreated. Chondroitin sulfate was detected using an anti-chondroitin sulfate antibody (CS-56) and goat anti-mouse immunoglobulin G (IgG) Alexa Fluor^®^ 488 secondary antibody (*green*). Nuclei were stained with DAPI (*blue*). **(b)** Plasmid red fluorescent protein (pRFP) was delivered to the left and right tibialis anterior (TA) muscles of C57Bl6 mice treated with either phosphate-buffered saline (PBS) or Cho ABC with or without EP. After 72 h, visualization of the reporter gene was performed by using the ProteinSimple imaging system. **(c)** Measurement of fluorescence intensity (FI) was accomplished using Adobe Photoshop. Animals per group = 3. Statistics were acquired with a Mann–Whitney test. **p = 0.0022.

The chondroitin sulfate ECM structure in the skeletal muscle represents a potential barrier acting against the efficient biodistribution of a drug in this tissue. With the aim of negating this barrier, animal tissues were treated with a chondroitin sulfate hydrolyzing enzyme, Cho ABC. The specific effect of Cho ABC on the muscle chondroitin sulfate ECM structure is depicted in Fig. 1a. A loss of chondroitin sulfate signal in female Hartley guinea pig muscle tissue sections treated with Cho ABC was observed. The effect of Cho ABC on the hyaluronan component of the ECM was limited (Supplementary Fig. S1).

To assess the effect on local gene expression after targeting chondroitin sulfate for disruption at the site of pDNA delivery, Cho ABC or PBS was injected into mouse TA muscles 30 min prior to delivering a pDNA encoding RFP with or without EP. All mice were monitored after treatment. There were no obvious additional visual signs of impedance in movement or discomfort in the EP + Cho ABC group compared to the EP + PBS group. Seventy-two hours after treatment, mice were sacrificed, hind limbs were dissected, and skin was removed. Visualization and measurement of the RFP intensity revealed reporter gene expression in the mouse muscle to be significantly enhanced in the Cho ABC + EP group (Fig. 1b and c). There was a 3.23-fold increase in the mean fluorescence intensity in the Cho ABC + EP group (3.02 × 10^5^ arbitrary units) muscles compared to the PBS + EP group (0.93 × 10^5^ arbitrary units). The findings indicate the administration of Cho ABC into the skeletal muscle of mice prior to delivery of pDNA EP enhances local gene expression. The use of Cho ABC independently of EP (Cho ABC –EP group) failed to increase the level of tissue gene expression significantly above background (PBS –EP group). Histological examination using H&E staining revealed no significant histopathological differences between muscles treated with Cho ABC or PBS (control) after the delivery of pDNA with EP (Supplementary Fig. S2).

### Enhanced gene expression levels in circulation after pDNA delivery into Cho ABC-treated muscles of mice

**Figure 2.**
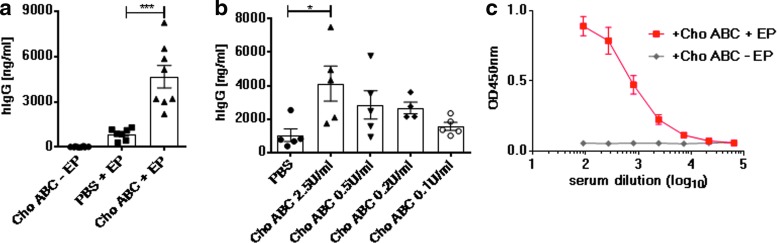
pDNA delivery into chondroitinase-treated muscles results in enhanced systemic gene expression in mice. **(a–c)** Mice were either intramuscularly treated with Cho ABC or with PBS 30 min prior to pDNA delivery with or without EP. Animals were bled on day 6, and serum human IgG (hIgG) levels were measured by enzyme-linked immunosorbent assay (ELISA). Animals per group = 8. **(b)** Doses of Cho ABC ranging from 0.1 to 2.5 IU/mL were tested. Animals per group = 5. **(c)** Binding of hIgG in the serum to the MERS CoV antigen was measured by ELISA. Graph depicts serum dilution binding curve on samples collected 6 days after pDNA delivery. Animals per group = 8. Statistics were acquired with Mann–Whitney tests. ***p < 0.0003; *p = 0.0317.

The study proceeded to assess whether a combination of EP with Cho ABC leads to enhanced gene expression by monitoring expressed circulating protein levels in the serum. Mouse TA muscles were treated with the enzyme (2.5 IU/mL) or PBS 30 min before intramuscular delivery of a plasmid encoding a hIgG molecule targeting Middle East respiratory syndrome (MERS) Co-V antigen with or without EP. On day 6 after treatment, the levels of hIgG were measured in the serum by enzyme-linked immunosorbent assay (ELISA; Fig. 2a). Gene expression analysis was performed no later than day 6, as a host immune response was elicited in the animals against the xenogeneic protein product (hIgG). This host response was associated with blunted gene expression at later time points. Pretreatment of the muscle delivery site with Cho ABC (Cho ABC + EP group) resulted in a 5.5-fold increase in hIgG serum levels (4,668.05 ng/mL) compared to the PBS + EP group (*M* = 856.02 ng/mL) on day 6 after treatment. Negligible serum levels of hIgG were observed in the group pretreated with Cho ABC followed by pDNA delivery without EP. The study proceeded to determine an optimal concentration of Cho ABC to achieve robust gene expression. Four escalating doses of the enzyme starting at 0.1 IU/mL to 2.5 IU/mL were administered into the TA muscle of Balb/c mice 30 min before the delivery of pMERS with EP (Fig. 2b). Analysis of serum hIgG levels revealed 2.5 IU/mL was associated with the highest levels of hIgG detected in the serum. Further study of a higher dose of Cho ABC (5.0 IU/mL) did not reveal a significant difference in the associated gene expression compared to the 2.5 IU/mL dose. *In vivo* production of a functional antigen-binding antibody was confirmed by a MERS CoV antigen-binding ELISA (Fig. 2c). In summary, the ECM disrupting enzyme Cho ABC can be applied to EP-assisted intramuscular pDNA delivery protocols to increase both local and systemic levels of the encoded protein.

### Cho ABC formulation enhances pDNA-encoded protein expression in NZW rabbits

**Figure 3.**
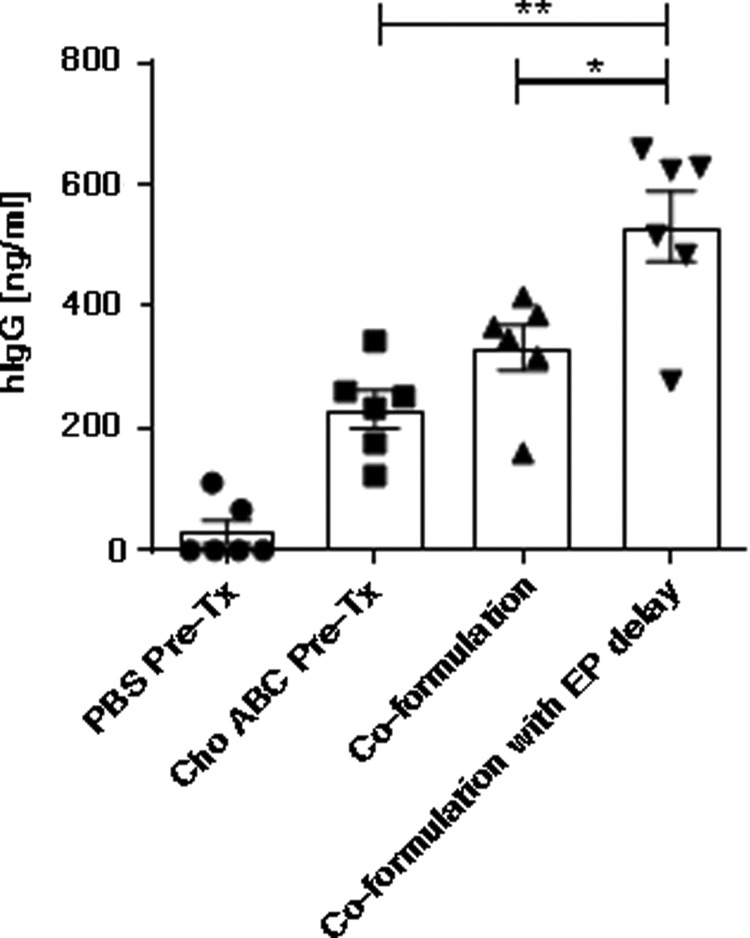
Cho ABC formulation enhances pDNA-encoded protein expression in New Zealand White rabbits. In control groups, muscles were pretreated with either Cho ABC (Cho ABC Pre-Tx) or PBS (PBS Pre-Tx) 30 min prior to pDNA delivery and EP. Co-formulated Cho ABC/pDNA was injected into the left rabbit quad muscle, and EP was initiated either immediately (co-formulation) or 1 min after injection (co-formulation with EP delay). The graph represents the levels of serum hIgG (ng/mL) measured by ELISA 5 days after pDNA delivery. Animals per group = 6. Statistics were acquired using Mann–Whitney tests. **p = 0.0043; *p = 0.0411.

The effects of Cho ABC in a pDNA gene-delivery protocol were assessed in a more clinically relevant surrogate animal model: the NZW rabbit. The NZW rabbit is considered a large-animal model, and is phylogenetically closer to primates and humans than mice are.^27^ The rabbit hind-limb skeletal muscle is significant larger and more complex in structure than the murine muscle. Importantly, the size of the rabbit quad muscle is compatible with a clinical intramuscular EP device, the CELLECTRA^®^-5P. Employing a protocol that incorporates Cho ABC pretreatment of the muscle, this study demonstrated a 7.89-fold enhancement of systemic gene expression after EP-assisted pDNA delivery into a quad-muscle site pretreated with the enzyme (233.37 ng/mL) compared to PBS (29.59 ng/mL; Fig. 3). With the aim of developing a delivery protocol appropriate for use in the clinic, the study investigated the possibility of co-formulating pDNA with the enzyme, and thus removing the need for the muscle pretreatment step. There was no significant difference between serum hIgG levels measured in the enzyme pretreatment or co-formulation group, indicating co-formulation was a viable option (Fig. 3). To provide additional time for the enzyme to disrupt the tissue ECM and allow for optimal distribution of pDNA across the muscle tissue, it was hypothesized that an extension of the time gap between injection of the pDNA/Cho ABC co-formulation into the tissue and the initiation of the EP pulsing may be advantageous. The study tested whether applying a 60 s gap (standard gap is 15–20 s) between injection and EP was advantageous. Significantly enhanced serum hIgG levels were observed in the co-formulation 60 s gap group (531.96 ng/mL) compared to the co-formulation standard group (332.48 ng/mL; Fig. 3). Additionally, the study tested whether a combination of two ECM-disrupting enzymes targeting different GAGs—hyaluronan and chondroitin sulfate—could act synergistically to enhance *in vivo* gene expression further. The formulation of Hylenex (human recombinant hyaluronidase) and Cho ABC with pDNA did not lead to a further increase in systemic gene expression (Supplementary Fig. S3).

### Stability of the pDNA/Cho ABC co-formulation

**Figure 4.**
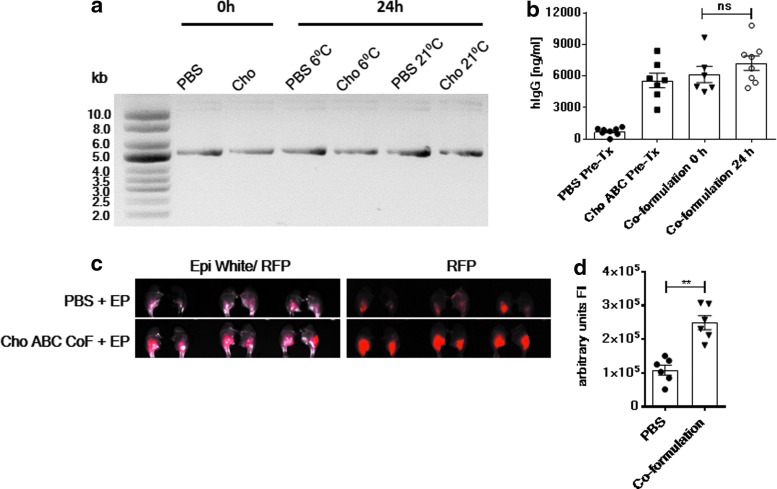
Short-term stability of the pDNA/Cho ABC co-formulation. **(a)** Agarose gel electrophoresis was run on pDNA samples stored in PBS only or Cho ABC (2.5 IU/mL) for 0 or 24 h at 6°C and 21°C. Ladder: supercoiled DNA ladder 2–10 kb (NEB). **(b)** Balb/c mice received a Cho ABC/pDNA co-formulation stored for 0 h (co-formulation 0 h) or 24 h (co-formulation 24 h) at 6°C into their left TA muscle followed by EP. Control groups received pDNA into their either Cho ABC (Cho ABC Pre-Tx) or PBS (PBS Pre-Tx) pretreated muscles. Blood samples were taken on day 6 after pDNA delivery, and hIgG levels were measured by ELISA. Statistics were acquired using a Mann–Whitney test. **(c)** RFP expression was analyzed on left and right TA muscles of C57Bl/6 mice administered with pRFP formulated with Cho ABC or PBS that had been stored for 24 h at 6°C. Reporter gene expression 72 h after pDNA administration was visualized by using an imaging system (ProteinSimple). **(d)** Measurement of FI was accomplished by Adobe Photoshop. Animals per group = 3. Statistics acquired using a Mann–Whitney test. **p = 0.0022.

The study proceeded to assess whether upon co-formulation pDNA remained structurally stable and the enzyme retained its activity. Within the scope of this study, only short-term storage was analyzed. Short-term (24 h) storage of the co-formulation under the influence of two different temperatures (6°C and 21°C) revealed no observable pDNA fragmentation after agarose gel electrophoresis compared to no storage (0 h; co-formulation immediately before analysis; Fig. 4a). Next, the study investigated whether 24 h storage at 6°C impacted the *in vivo* gene expression compared to “at site” formulation (pDNA and Cho ABC mixed 1 min before injection; 0 h). *In vivo* delivery of the pDNA/Cho ABC co-formulation stored for 24 h at 6°C to the TA muscle in Balb/c mice was associated with a 9.56-fold increase in gene expression (7,223.05 ng/mL) in comparison to the pDNA/PBS formulation group (755.16 ng/mL; Fig. 4b). No significant differences in gene expression were observed between the Cho ABC pretreatment, at site (0 h, 6,148 ng/mL), and 24 h co-formulation groups (7,223 ng/mL). The striking enhancement of local gene expression was also conserved with the pDNA/Cho ABC co-formulation stored for 24 h at 6°C (Fig. 4c and d). Significantly enhanced RFP expression levels were observed in the muscle of the mice administered the pDNA/Cho ABC co-formulation (2.49 × 10^5^ arbitrary fluorescence intensity units) compared to the pDNA/PBS co-formulation control group (1.08 × 10^5^ arbitrary fluorescence intensity units).

### Cho ABC formulation increases the immunogenicity of a DNA vaccine

**Figure 5.**
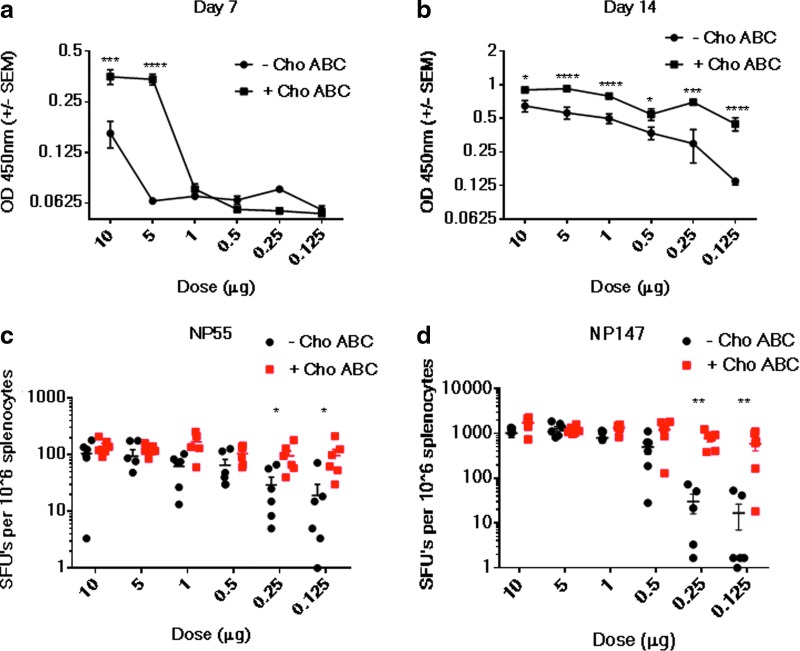
Host immune responses are augmented in a DNA vaccination protocol employing Cho ABC formulation and EP. pDNA influenza NP vaccine (pNP) was co-formulated with (+Cho ABC) or without (–Cho ABC) Cho ABC. Co-formulations containing pDNA concentrations ranging from 0.125 to 10 μg were administered to the left TA muscle of Balb/c mice followed by EP. To assess the humoral response elicited by the vaccine, peripheral blood was collected on day 7 **(a)** and day 14 **(b)** after pDNA delivery. Serum IgG binding to NP antigen was evaluated by ELISA (optical density = 450 nm). Number of animals per group = 6. Statistics were acquired using Mann–Whitney tests. *p < 0.05; ***p < 0.001; ****p < 0.0001. The CD4+ and CD8+ T-cell responses (spot-forming units) to NP55-69 **(c)** and NP147-155 **(d)** peptide epitopes, respectively, were measured by an interferon gamma enzyme-linked immunospot assay performed 14 days after pNP immunization on splenocytes. Statistics were acquired using Mann–Whitney tests. *p < 0.05; **p < 0.01.

The study assessed the versatility of the application of Cho ABC in a gene-delivery protocol in which a DNA vaccine was delivered. The humoral and cellular immune responses elicited after a single dose of an influenza NP antigen pDNA (pNP) vaccine formulated with or without Cho ABC delivered intramuscularly with EP were measured. It was hypothesized that the enhanced gene expression associated with Cho ABC formulation would permit dose sparing. To test this, groups of BALB/c mice received doses of pNP vaccine ranging from 0.125 to 10 μg formulated with Cho ABC or PBS. To measure the humoral response to the vaccine, serum samples were obtained from the mice on days 7 and 14. Serum levels of NP antigen-binding antibodies were measured by ELISA. On day 7, accelerated seroconversion was observed at doses of 5 and 10 μg in the groups of mice that received the pNP vaccine formulated with Cho ABC (Fig. 5a). A clear dose sparing effect was detected in the Cho ABC group on day 14, with significantly elevated antibody responses detected at both high and low doses (Fig. 5b). The antigen-specific cellular responses to the class II and class I major histocompatibility complex–restricted NP epitopes NP55-69 and NP147-155, respectively, were analyzed by an IFN-γ ELISpot (Fig. 5c and d). An equivalent cellular immune response was observed between the Cho ABC and PBS formulation groups immunized with pNP doses >0.5 μg. However, at lower doses (0.25 μg to 0.125 μg), CD4+ T-cell responses to NP55-69 (Fig. 5c) and CD8+ T-cell responses to NP147-155 (Fig. 5d) were significantly enhanced in mice immunized with pDNA formulated with Cho ABC. The data suggest the employment of Cho ABC in pDNA vaccine delivery protocols may allow for vaccine dose sparing.

## Discussion

pDNA-based gene delivery is a powerful platform with the potential to address unmet medical needs in the fields of infectious disease, oncology, and gene replacement therapies. Here, an enhanced pDNA delivery protocol to increase the levels of *in vivo* protein production generated by this platform is delineated.

While EP enhances the passage of pDNA across the cell membrane barrier, another significant obstacle inhibiting optimal gene transfer is the complex branch-like structure of the ECM. The ECM may impede the distribution of pDNA across the tissue and the proximity of pDNA to the myocytes, negatively impacting the number of cells available for transfection. Previous studies with the ECM-disrupting enzyme hyaluronidase have demonstrated that the temporary removal of this barrier increased pDNA diffusion in the tissue, increasing cell exposure and transfection efficiency when combined with EP-assisted delivery.^23,24,28^

Here, the enzyme Cho ABC was tested with the aim of disrupting the ECM barrier. The bacterial-derived Cho ABC was selected, since this enzyme is a clinical-grade product that is currently being investigated in a Phase III clinical trial for the treatment of radicular leg pain due to lumbar disc herniation (NCT 02421601). The proposed mechanism is that Cho ABC catalyzes the transient hydrolysis of chondroitin sulfate. Indeed, the data confirm that Cho ABC degrades chondroitin sulfate in the skeletal muscle ECM. Disrupting chondroitin sulfate in the muscle may lead to increased tissue permeation and accessibility to the myocytes. A similar mechanism has been suggested for hyaluronidase,^23,24^ a licensed drug-delivery enhancer. To differentiate the ECM degradation mechanisms of Cho ABC and hyaluronidase, hyaluronan was visualized after incubation of muscle tissue with either hyaluronidase or Cho ABC. Since hyaluronan was not significantly affected by the addition of Cho ABC (Supplementary Fig. S1), these data further confirm that Cho ABC specifically drives the degradation of chondroitin sulfate in the muscle tissue. Previous studies have reported that Cho ABC processes chondroitin sulfate and dermatan sulfate at robust rates, with a clear preference for chondroitin 4-sulfate (C4S) and chondroitin 6-sulfate.^29,30^ The catalytic efficiency was reported to be 20-fold higher for C4S compared to hyaluronan.^29^ Similarly, hyaluronidases have been considered to hydrolyze chondroitin sulfate. However, hyaluronidases digest chondroitin sulfate more slowly than hyaluronan, and its preferred substrate is hyaluronan rather than chondroitin sulfate,^31–33^ representing the distinct mechanisms of Cho ABC and hyaluronidase. In consideration of this, this study investigated whether the combined use of hyaluronidase and Cho ABC led to further enhancement of gene expression. However, muscle treatment with both enzymes failed to enhance local or systemic gene expression over the use of the enzymes individually (Supplementary Fig. S3).

Importantly, the present study demonstrated a potential clinical relevant protocol for the use of Cho ABC in the delivery of pDNA to the muscle with *in vivo* EP by scaling the technology up to the rabbit model and using a pDNA and enzyme co-formulation to negate the need for a pretreatment step. The rabbit is significantly larger than the mouse (2.5 kg vs. 20 g), possesses a greater blood volume (140 mL vs. 1.2 mL), has significant larger muscles, and is phylogenetically closer to primates than mice.^27^ Additionally, the rabbit is a favorable preclinical animal model for this EP delivery protocol, since the size of the muscle allowed the CELLECTRA^®^-5P device to be used, which is currently applied in the clinic. Thus, the rabbit model was employed to develop a clinically relevant delivery protocol. To this purpose, further optimization of the formulation was needed to address the inadequacy of the pretreatment protocol. Administration of a single vial formulation would be more comfortable for the patient, technically simpler for the clinician, and time-saving for both parties. After development of an appropriate co-formulation protocol, initially equal systemic gene expression (serum hIgG levels) was observed when compared to the standard pretreatment protocol. However, enhanced serum hIgG levels were observed when the time lag between formulation injection and initiation of EP was extended to 60 s. Currently, it is predicted that this 60 s time lapse may allow for enzyme saturation and a level of ECM disruption, which leads to increased pDNA interaction with myocytes and bolus dispersion across an area compatible with the EP field.

The study proceeded to test the stability of the co-formulation. pDNA in suitable buffer remains stable through long-term storage at room temperature and does not require a cold chain for distribution.^34,35^ The study evaluated the stability of pDNA upon co-formulation with Cho ABC. This approach demonstrated that pDNA and enzyme can be stored for at least 24 h at 6°C or 21°C without impacting *in vivo* expression of the gene (Fig. 4). These data provide promising early indications concerning storage conditions and the potential shelf life of such formulations. Further studies to determine the long-term storage potential are currently ongoing.

To demonstrate the versatility of this technology, the study evaluated whether Cho ABC could be employed to enhance the immunogenicity associated with DNA vaccine protocols. The results indicated that the enhanced antigen gene expression associated with the Cho ABC delivery protocol augmented the immune response in a dose-dependent manner. This suggested Cho ABC formulation will permit the antigen threshold required for the elicitation of an immune response to be achieved with lower pDNA doses. While this threshold may be achieved without the use of Cho ABC formulation in small-animal models such as mice by using high pDNA doses compared to body weight (mg/kg), in large-animal models and humans, these dose-to-weight ratios are more difficult to reach. The use of Cho ABC formulations may allow the higher antigen threshold in larger animals to be reached. This is being actively investigated.

Collectively, the results from this preclinical study support the continued advancement of a pDNA-based gene-delivery protocol employing Cho ABC formulation. Cho ABC formulation may provide an excellent tool to develop pDNA-based delivery protocols to address a wide range of medical needs in the field of infectious disease, oncology, and protein replacement therapies.

## Supplementary Material

Supplemental data

Supplemental data

Supplemental data
