# Supplementary material for: Active Immunoprophylaxis and Vaccine Augmentations Mediated by a Novel Plasmid DNA Formulation
Source: Hum Gene Ther. 2019 Apr 4;30(4):523–33. doi: 10.1089/hum.2018.241 (PMC6479233; doi:10.1089/hum.2018.241)
Supplement: Supplemental data [file Supp_Fig1.pdf]

Supplementary Data

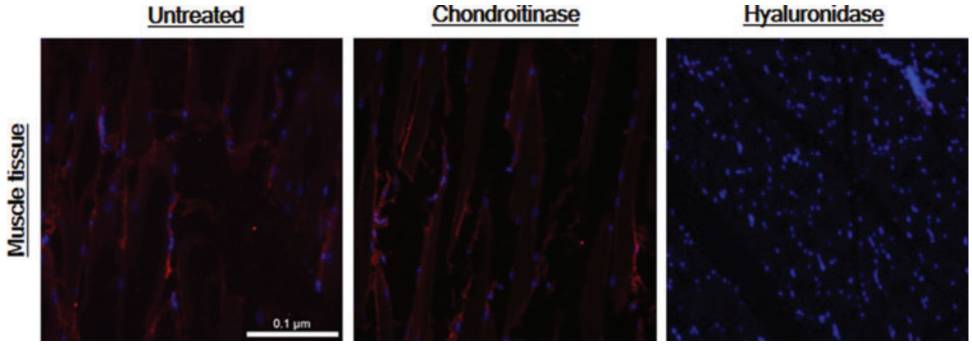

Supplementary Figure S1. Chondroitinase does not target hyaluronan in murine muscle tissue. Left TA muscles of C57Bl6 mice were dissected and immediately transferred into 4% paraformaldehyde, and 9 μm sections were incubated with either 2.5 IU/mL Cho ABC or 150 IU/mL hyaluronidase (3 h at 37°C) or were left untreated. Hyaluronan was detected using biotinylated HABP and streptavidin, with Alexa Fluor® 555 secondary antibody (*red*). Nuclei were stained with DAPI (*blue*).
