# Supplementary material for: Active Immunoprophylaxis and Vaccine Augmentations Mediated by a Novel Plasmid DNA Formulation
Source: Hum Gene Ther. 2019 Apr 4;30(4):523–33. doi: 10.1089/hum.2018.241 (PMC6479233; doi:10.1089/hum.2018.241)
Supplement: Supplemental data [file Supp_Fig2.pdf]

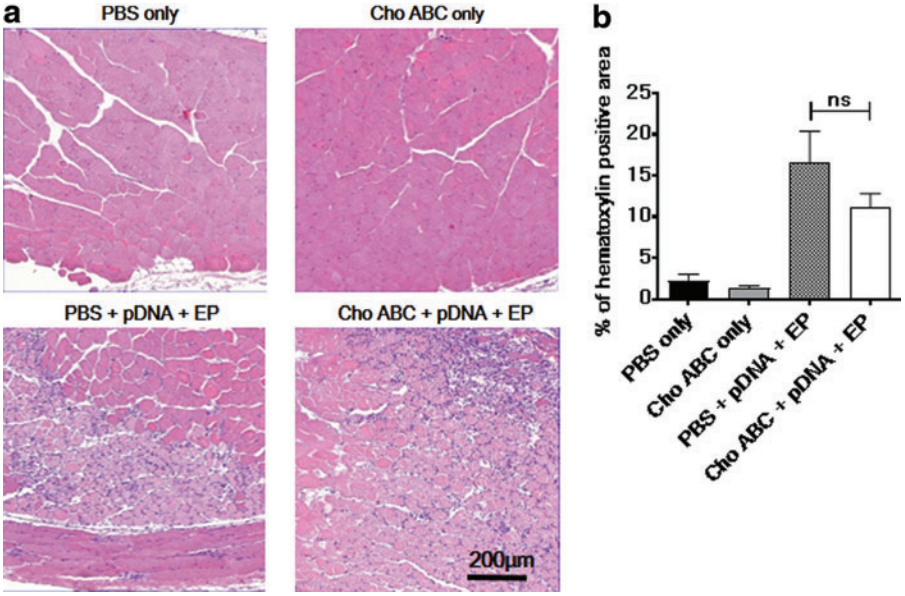

Supplementary Figure S2. Representative histopathology of murine TA muscle performed by H&E staining. **(a)** *Top panels* show TA muscle tissue treated with either PBS only (control) or Cho ABC (2.5 IU/mL) only. Muscles pretreated with PBS or Cho ABC before pDNA delivery with EP are presented in the *bottom panels*. Results were analyzed by a slide scanner and CaseViewer software (3DHISTECH). Scale = 200 μm. **(b)** Quantification of each group was performed using ImageJ. Statistics were acquired using Mann–Whitney tests.
