# Supplementary material for: Active Immunoprophylaxis and Vaccine Augmentations Mediated by a Novel Plasmid DNA Formulation
Source: Hum Gene Ther. 2019 Apr 4;30(4):523–33. doi: 10.1089/hum.2018.241 (PMC6479233; doi:10.1089/hum.2018.241)
Supplement: Supplemental data [file Supp_Fig3.pdf]

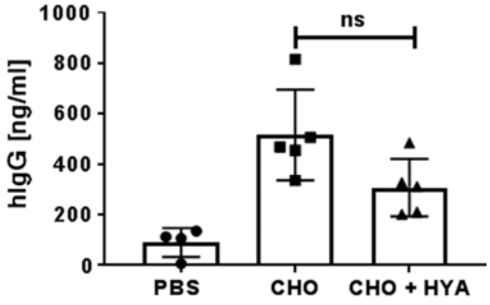

Supplementary Figure S3. Combination of Cho ABC with hyaluronidase (Hylenex) does not lead to further enhancement of pDNA-encoded protein expression in NZW rabbits. Rabbits were treated with PBS + pDNA, Cho ABC + pDNA, or Cho ABC + Hylenex + pDNA. Formulations were injected into the left rabbit quad muscle, and EP was initiated 1 min after injection. The graph represents the levels of serum hIgG (ng/mL) measured by ELISA 5 days after pDNA delivery. Animals per group = 4–5. Statistics were acquired using Mann–Whitney tests.
